# Linking shape and clinical outcomes: Statistical shape model of Bidirectional Glenn connection in congenital single ventricle patients

**DOI:** 10.1371/journal.pdig.0001611

**Published:** 2026-08-18

**Authors:** Maria Arrese-Zubizarreta, Naiara Rodriguez-Florez, Raphael Sivera, Liam Swanson, Claudio Capelli, Syed Faaz Ashraf, Jan Lukas Bruse, Tain-Yen Hsia, Silvia Schievano

**Affiliations:** 1 Institute of Cardiovascular Science, University College London and Great Ormond Street Hospital for Children, London, United Kingdom; 2 Department of Mechanical Engineering and Materials, Universidad de Navarra, TECNUN Escuela de Ingenieros, San Sebastian, Spain; 3 Ikerbasque, Basque Foundation for Science, Bilbao, Spain; 4 University of Pittsburgh, Pittsburgh, Pennsylvania, United States of America; 5 Fundación Vicomtech, Basque Research and Technology Alliance (BRTA), San Sebastian, Spain; 6 Heart Center at Arnold Palmer Hospital for Children, Orlando, Florida, United States of America; University of Pennsylvania, UNITED STATES OF AMERICA

## Abstract

In patients with congenital single ventricle physiology, the Bidirectional Glenn procedure is a critical intermediate step toward Fontan circulation. Despite technical advances, morbidity and mortality remain significant. While left pulmonary artery (LPA) stenosis is a recognized complication, the broader impact of 3D anatomical variations on outcomes remains poorly understood. We aimed to characterize morphological variability of the superior cavopulmonary connection (SCPC) following the Bidirectional Glenn and assess its relationship with clinical outcomes. Statistical Shape Modeling (SSM) was applied to cardiovascular magnetic resonance images from 29 single ventricle patients following Bidirectional Glenn, whose SCPC showed no clinically important SCPC or pulmonary arterial stenosis. A population-based 3D anatomical template was generated using the Deformetrica framework. Patient-specific shape deformations were quantified and correlated with clinical metrics—including SCPC pressure, oxygen saturation, intensive care unit (ICU) stay, and total hospital stay—via Partial Least Squares (PLS) regression. Associations were compared to correlations with conventional morphological parameters. SSM revealed 3D shape features significantly associated with SCPC pressure and ICU stay (p < 0.01). Lower SCPC pressure correlated with straighter, wider LPA geometry. In contrast, narrower—but without overt stenosis—LPA morphologies were associated with worse outcomes. Conventional geometric metrics did not show such correlations. No association was observed between SCPC shape and total hospital stay. This study provides a novel 3D shape-based characterization of SCPC anatomy in single ventricle patients post Bidirectional Glenn surgery and its link to outcomes. SSM-derived morphological biomarkers offer potential for improved risk stratification and surgical planning in congenital heart disease.

## Author summary

Patients with congenital single ventricle heart defects undergo a series of complex surgeries, including the Bidirectional Glenn procedure, which reroutes blood flow toward the lungs. Despite surgical advances, complications and variability in outcomes remain significant. One known issue is narrowing of the left pulmonary artery (LPA), but the impact of overall three-dimensional (3D) heart anatomy on clinical outcomes is not well understood. In this study, we used cardiovascular magnetic resonance imaging from 29 patients to create the first 3D statistical shape model of the superior cavopulmonary connection (SCPC) after the Bidirectional Glenn procedure. This model allowed us to quantify individual variations in SCPC anatomy and examine their relationship with clinical outcomes such as SCPC pressure, oxygen saturation, and length of stay in the intensive care unit. We found that subtle differences in LPA geometry—such as straighter, wider vessels—were associated with better short-term outcomes, while narrower but non-stenotic vessels were linked to higher pressures and longer ICU stays. Conventional measures of vessel size and angles did not reveal these associations. Our work provides a digital framework for understanding anatomical variability, offering potential for improved risk assessment, personalized surgical planning, and future research in congenital heart disease.

## Introduction

Congenital heart defects (CHDs) represent the most prevalent category of birth anomalies and a major global health concern, standing as a leading cause of mortality and premature death during infancy and childhood [1–3]. Despite significant regional variations in awareness and medical resources, the global incidence of CHD has shown a notable increase, particularly in developing nations, contributing substantially to the burden of pediatric hospitalizations [1]. While recent advancements in surgical techniques and catheter-based interventions have remarkably improved survival rates into adolescence and adulthood, CHDs still necessitate lifelong specialized follow-up [4]. However, longitudinal data regarding lesion-specific mortality remains limited, underscoring the need for more precise tools to monitor long-term clinical outcomes in complex physiology [5].

Single Ventricle (SV) physiology encompasses a spectrum of congenital heart defects (CHDs) characterized by the presence of only one functional ventricular chamber rather than separate right and left ventricles in normal hearts [6]. These conditions represent major clinical challenges due to their anatomic and physiologic complexity, universal fatality without treatment, and the necessity for staged surgical interventions, beginning in few days of life, to maintain viable cardiopulmonary circulation. Despite significant refinements in surgical approach and perioperative management, patients undergoing these procedures continue to face disproportionately high rates of morbidity and mortality compared to those with less complex congenital heart defects [7]. A multitude of risk factors has been shown to influence long-term success in this population. Longitudinal data from large cohorts indicate that hemodynamic and patient-specific variables—such as elevated pulmonary artery pressure, younger age at the time of intervention, and significant pulmonary artery distortion—are strong predictors of adverse outcomes [8]. Furthermore, the timing of diagnosis and the presence of valvular dysfunction play critical roles; for instance, prenatal detection has been linked to improved early survival following the bidirectional cavopulmonary connection [9], whereas moderate-to-severe atrioventricular valve regurgitation remains an independent predictor of mortality, particularly in complex cases such as heterotaxy [10]. Collectively, these findings underscore that while identifying modifiable clinical and surgical risk factors has been essential, further research into optimizing patient-specific management is crucial for improving the prognosis of this complex population. In cases of SV associated with reduced pulmonary blood flow, initial palliation usually involves placement of a modified Blalock-Taussig shunt between the systemic and pulmonary circulations, thus putting the systemic and pulmonary circulations in parallel, instead of the normal in-series arrangement [11]. Other defects associated with too much pulmonary blood flow, a band is placed on the main pulmonary artery to achieve a balance between systemic and pulmonary circulations. Finally, in patients with Hypoplastic left heart syndrome (HLHS), a complex neonatal operation called Norwood procedure is employed, which incorporated a modified Blalock-Taussig or right ventricle-to-pulmonary artery shunt to provide pulmonary blood flow [12]. By placing the systemic and pulmonary circulations in parallel, any of the Stage 1 procedures obligates the single ventricle to the works of both systemic and pulmonary circulations, when normally each has its own dedicated ventricle. Following the first stage operations, patients typically undergo a second stage operation, at around 5–6 months of age, to create a superior cavopulmonary connection (SCPC) and finally a third stage operation at around 2–3 years age to achieve the total cavopulmonary connection or Fontan circulation.

**Fig 1:**
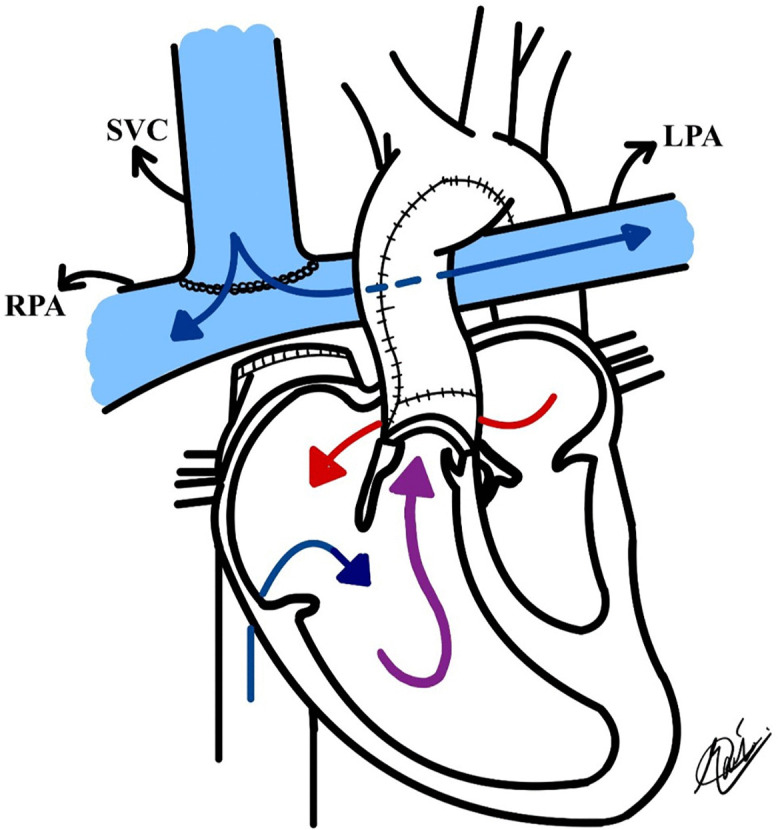
This figure illustrates the heart’s anatomy following the second-stage Bidirectional Glenn procedure. In this operation, oxygen-poor blood enters through the superior vena cava, and thanks to the Superior Cavopulmonary Connection (SCPC) (highlighted in blue) created during the surgery, the blood is directed straight to the lungs via the pulmonary arteries.

The Bidirectional Glenn procedure (Fig 1), originally described five decades ago [13] remains a critical component of this pathway by establishing a SCPC circulation. It involves redirecting superior vena cava (SVC) blood flow directly to the pulmonary arteries via end-to-side anastomosis of native-to-native tissues, thereby bypassing the heart and improving the efficiency of pulmonary perfusion without an non-growing artificial shunt or ever-restrictive pulmonary artery band. Moreover, the SCPC relieves the single ventricle of pulmonary blood flow work, thus facilitating later Fontan circulation while allowing for somatic growth. In both the SCPC or TCPC circulations, the pulmonary circulation work responsible for the return of deoxygenated venous blood to the lungs is carried out *without* the assistance of a ventricular power, leading to obligatory higher venous pressures than normal. However, elevated venous pressures further from the optimal SCPC or TCPC pressures are harbingers of failing pulmonary circulation and poor patient outcomes.

Despite its widespread adoption and refinement, the Bidirectional Glenn procedure remains associated with significant inter-patient variability in post-operative vascular anatomy, which may influence long-term outcomes [14]. Emerging evidence suggests that the configuration of the SVC-to-PA junction, as well as geometry-related factors such as vessel caliber, curvature, and asymmetry, can affect flow patterns, remodeling, and future Fontan feasibility. In particular, there is concern that traditional Glenn configurations may impair growth of the central pulmonary artery or promote thrombosis, thereby compromising long-term function [15,16]. Current literature from the last five years confirms that while the bidirectional Glenn (BDG) procedure achieves clinically acceptable survival rates—with mortality ranging between 3.3% and 3.8% [17,18]—postoperative morbidity remains a significant challenge. Recent longitudinal cohorts have reported complication rates as high as 57.4%, with infectious processes being the most prevalent [18]. Furthermore, established predictors of adverse outcomes have been identified, including right ventricular dominance, elevated pulmonary vascular resistance, and protracted intensive care requirements [19]. Innovative surgical strategies, such as performing a direct BDG without prior pulmonary artery banding, have also shown promise in improving survival for specific patient subsets [20].

However, despite these advances in identifying clinical and physiological risk factors, there is a notable absence of data regarding the influence of global 3D morphology on these outcomes. While recent studies demonstrate that BDG surgery achieves acceptable results through the management of known variables, they do not address the potential of statistical shape modeling (SSM) to quantify the impact of complex anatomical remodeling. Consequently, how specific 3D vascular configurations independently affect clinical performance remains largely unexplored.

To date, most clinical evaluations of post-Glenn anatomy have relied on qualitative or simplified 2D measurements—diameters, angles, and flow profiles—which provide only limited insight into the complex, high-dimensional nature of vascular remodeling in this setting [21,22]. While computational fluid dynamics models have been applied to small patient cohorts to estimate flow fields and predict Fontan hemodynamics [16], these studies have generally treated anatomical geometry as static or idealized, and few have systematically investigated how clinical variables shape the actual 3D vascular architecture following Bidirectional Glenn.

There is therefore a growing need for data-driven, computational frameworks capable of capturing and analyzing anatomical variability across patient cohorts. Statistical shape modelling (SSM) combines elements from geometry, statistics, and computer science, and is increasingly used across various domains of medical image analysis [23–28]. SSM enables the quantification of shape variability in anatomical structures using 3D meshes derived from clinical imaging data. These models have been successfully applied to analyze the aorta [23,24,29], cerebral ventricles [30], and left ventricles in the context of LVAD-related thrombosis risk [28]. By representing complex vascular anatomy as parameterized shape vectors, SSM allows for objective comparison, dimensionality reduction, and correlation with clinical or functional outcomes.

In this work, we present the first application of an SSM framework to characterize the SCPC following the Bidirectional Glenn procedure. Using automated, landmark-free 3D shape modeling, we investigate how post-operative anatomy varies across patients, and whether this variation is associated with clinical parameters. Our approach enables a computational analysis of vascular shape in this unique physiological setting—an essential step towards developing predictive models that support surgical planning and patient-specific risk stratification in congenital heart disease.

## Materials and methods

### Patient population and clinical data

We analyzed routine post-Glenn cardiovascular magnetic resonance (CMR) imaging data from 29 single ventricle (SV) patients. The average age at the time of the second-stage Glenn procedure was 0.62 ± 0.55 years. CMR scans were performed as part of routine follow-up at GOSH before the Fontan surgery, at an average of 2.9 ± 0.8 years after the Bidirectional Glenn procedure. CMR imaging was conducted on a 1.5 T Avanto MR scanner (Siemens Medical Solutions, Erlangen, Germany). To ensure high anatomical fidelity for the Statistical Shape Model (SSM), a 3D balanced steady-state free precession (bSSFP) whole-heart sequence was used, with acquisition performed during the mid-diastolic rest period. Images were reconstructed with a 1.3 mm isotropic voxel resolution, providing a consistent spatial definition across the cohort. All patients were managed and imaged at this single-center institution. None of the patients had hemodynamically significant obstruction, nor any stents or shunts that could interfere with the CMR results.

Among the 29 patients, there were 10 different underlying conditions across the entire patient cohort (S1 Appendix), which included a total of 16 patients who had undergone initial surgical repair with the Norwood procedure.

### Ethics statement

Ethical approval was obtained for the use of image data for research purposes, and informed consent was given by all patients’ parents or legal guardians of each participant under 18 years of age. The collection of the private dataset used in this study conformed to the principles of the Declaration of Helsinki and was approved by the UK National Health Service, Health Research Authority, Research Ethics Committee and written informed consent was obtained for all subjects (Code: 06/Q0508/124).

### Medical Image processing

Superior cavopulomary connection pressure (pSCPC) and oxygen saturation (SaO_2_) were measured prior to CMR imaging and before undergoing the third-stage Fontan procedure. Clinical recovery variables of the Glenn procedure were also recorded, including days spent in the intensive care unit (LICU) and total length of hospital stay (LOHS) in days following the Bidirectional Glenn procedure. Body surface area (BSA) of each patient was calculated using the Haycock [31] formula, and morphological parameters were indexed to BSA where appropriate.

**Fig 2:**
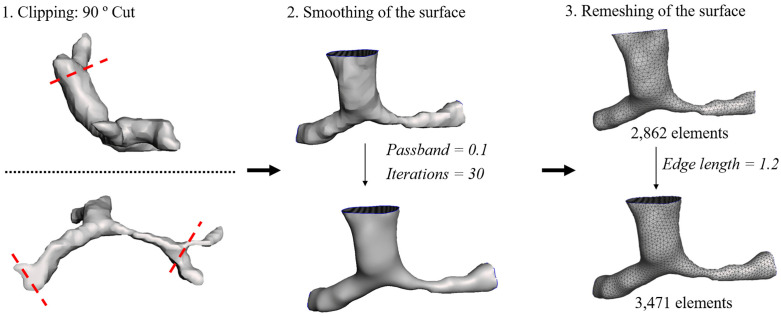
Pipeline of the processing of medical images of the Superior Cavopulmonary connection complex prior to SSM: clipping, smoothing and remeshing of the surface.

**Fig 3:**
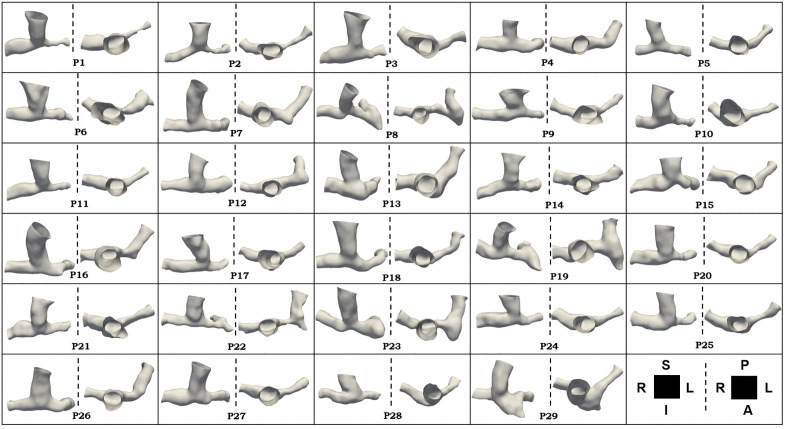
3D models of superior cavopulmonary connection complex of 29 patients post Bidirectional Glenn procedure.

The anatomical structures of interest consisted of the right and left pulmonary arteries (RPA and LPA), as well as the superior vena cava (SVC) (Fig 3). The structures were segmented from CMR data and converted to 3D computational surface meshes using ITK-SNAP software [32], following protocols described in [33–35]. The image post-processing workflow is summarized in Fig 2. The first preprocessing step consisted of generating open inlets and outlets by clipping the surfaces using Meshmixer [36] software. Clipping was applied at the bifurcation point between the SVC and both pulmonary arteries. Care was taken to ensure that the cuts were made perpendicular to the vessel surfaces, resulting in three standardized openings per subject. Subsequently, surface smoothing and remeshing were conducted using custom Python scripts, incorporating various functions from the Vascular Modeling Toolkit (VMTK) [37,38]. Surface smoothing was performed using the *vmtksurfacesmoothing* function, with a passband of 0.1 and 30 fixed iterations. Remeshing was then applied using the *vmtksurfaceremeshing* function, with a target edge length of 1.2 mm for boundary conditions. The resulting anatomical structures for all patients are shown in (Fig 3).

### Statistical shape modelling for template computation

A statistical shape modeling (SSM) [24] approach was employed to quantitatively analyze the 3D anatomical variability in a cohort of 29 post-Glenn SV. This analysis was conducted using an automated, landmark-free computational pipeline based on the surface meshes derived from CMR. No manual annotation or traditional morphological measurements (e.g., diameters, angles) were required at any stage.

Surface meshes were processed using *Deformetrica* (www.deformetrica.org) [39], an open-source software based on the Large Deformation Diffeomorphic Metric Mapping (LDDMM) framework. In this setting, anatomical surfaces are represented as *mathematical currents* [40], which are surrogate representations of shapes, and diffeomorphic deformations between shapes are parameterized via control points and associated momentum vectors. The method allows for high-fidelity deformation modelling in 2D or 3D space, and is well-suited for topologically consistent, smooth anatomical domains where landmarking is challenging [41,42].

The framework computes a population-based anatomical *template shape T*, or mean shape, via simultaneous registration of all subjects in the cohort. For each subject, a corresponding set of deformation function $\emptyset_{i}$ is calculated i∈[1, 29], describing the transformation needed to morph the template geometry into the patient-specific shape. Each patient SCPC is therefore expressed as a deformation towards the template shape $\emptyset_{i}(T)$ and numerically parameterized by a set of deformation vectors $\beta_{i}$ generated by Gaussian kernels, as detailed in [39,42]. All these deformation vectors constitute a deformation matrix *M* (Eq. 1), which captures morphological variation across the cohort in a compact and data-driven manner [27].


$$\begin{array}{c} M=[\begin{array}{ccc} -- & \beta_{\text{1}}^{T} & --\\ -- & \ldots & --\\ -- & \beta_{\text{2}\text{9}}^{T} & -- \end{array}] \end{array}$$ (1)

**Fig 4:**
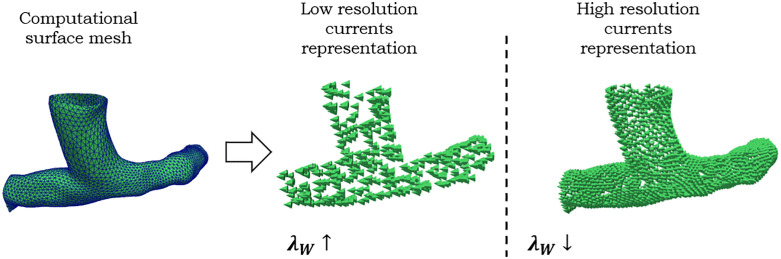
Differences in the Superior Cavopulmonary connection complex caused by changing the deformation resolution parameter $\lambda_{𝐖}$.

In order to define the deformation vectors, four key parameters must be set:

- The initial base geometry: The reference shape used to initiate the template construction (one of the shapes from Fig 3);
- Deformation resolution (λ_W_): Controls the spatial granularity of the deformation. Lower values allow for fine-grained shape matching at the risk of overfitting, whereas higher values promote smoother transformations with reduced sensitivity to subtle features (Fig 4);
- Stiffness (λ_V_): Determines the density of control points. Reduced stiffness yields a higher number of control points, increasing the deformation capacity at the cost of computational complexity and potential noise amplification;
- Noise level: Regulates the tolerance to residual differences between deformed templates and target shapes.

To ensure consistent deformation fidelity across patients, resolution (λ_W_) was initialized according to the surface area (A_surf_) of the smallest geometry in the cohort, as suggested by [29], and shown in Eq. 2:


$$\begin{array}{c} \lambda_{W}\;=\;\sqrt{(\text{0}.\text{0}\text{2}\text{5}A_{surf})\;} \end{array}$$ (2)

The deformation process was optimized by an iterative registration strategy based on the Iterative Closest Point (ICP) algorithm. This was used to align all input geometries to an approximate mean shape before computing the Deformetrica-based deterministic template. Three iterations of template computation were required to meet this convergence criterion. Once the final alignment was achieved, additional tests were performed to assess the influence of each parameter—initial guess, stiffness, resolution, and noise—on the resulting template. These tests were used to select the combination of parameters that produced the highest-quality template, based on the Akaike Information Criterion (AIC) [43] (Eq. 3) for model selection.


$$\begin{array}{c} AIC=-\text{2}Llh+\text{2}\cdot \text{3}\cdot N_{CP}\cdot (N_{S}+\text{1}) \end{array}$$ (3)

Where, *Llh* is the Log-likelihood, which is a measure of how well a particular model fits the data; *N*_*CP*_ is the number of control points (*N*_*CP*_ = 140); and *N*_*S*_ is the number of shape subjects (*N*_*S*_ = 29).

After identifying the optimal parameters —initial guess based on the average surface area subject (P4), stiffness λ_V_ = 8, resolution λ_W_ = 5, and noise = 1, a final run was conducted using the best combination, resulting in a refined population-level template (anatomical 3D mean shape of the post-Glenn SCPC) and a deformation matrix *M*. Detailed methods used to optimize and define these parameters are described in [24].

Validation of the computed template was performed through two complementary approaches. First, a geometric comparison was used to confirm that the global shape metrics of the template (volume, surface area, and diameters of the SVC and pulmonary arteries) closely match the cohort-wide mean values derived using the Vascular Modeling Toolkit (VMTK). Second, to assess the robustness of the template generation, a repeated random subsampling strategy was employed. In each of the 10 iterations, subjects were randomly excluded from the cohort (approximately 10% of the sample per iteration) ensuring that every subject was left out at least once across the full process. The resulting template shapes from each iteration were compared to the global template to verify morphological stability. This validation confirmed that the average anatomy remained consistent regardless of specific subject combinations.

### Partial least squares regression

The deformation matrix *M* served as the input for the quantification of 3D shape features most associated with relevant clinical parameters via partial least squares regression (PLS) [29]. PLS analysis enabled the extraction of *PLS shape modes*, which represent the dominant 3D features of SCPC complex most correlated with the clinical parameters of interest: Superior cavopulmonary connection (pSCPC) pressure, oxygen saturation (SaO_2_), length of ICU stay (LICU), and total length of hospitalization (LOHS). Prior to this analysis, variability in body size between subjects was accounted for by removing body surface area (BSA)-related shape effects through a preliminary PLS regression, as previously described in the literature [23,29].

Each patient shape was projected onto the respective PLS shape mode in order to obtain a *PLS shape vector* (scalar product between $\emptyset_{i}$ and PLS shape mode). Each shape vector serves as a quantitative representation of a particular shape mode: its entries indicate, for each subject, the degree to which the template must be deformed along the mode to best approximate that subject’s anatomy. In essence, the shape vector encodes both global and regional 3D shape features relevant to individual subjects and the associated response parameter. A subsequent standard correlation analysis between the PLS shape vector and the response variable quantifies the extent to which the shape features captured by the mode correspond to variations in the response. A perfect correlation would suggest that the derived shape mode precisely characterizes those shape patterns linked to low or high values of the response parameter (e.g., extremes in physiological measurements or outcomes) [24,29].

3D shape features most related with a chosen response parameter were visualized in Paraview [44] after morphing the shape template *T* along the direction of the corresponding PLS shape mode, ranging from −4 to +4 standard deviations relative to the template shape *T*. Such 3D models of the extreme cases were used to enhance the most relevant anatomical features related to higher pSCPC pressure, lower oxygen saturation, and higher duration of ICU or hospital stay, which are all considered detrimental for the success of the Bidirectional Glenn procedure.

### Morphological parameters

**Fig 5:**
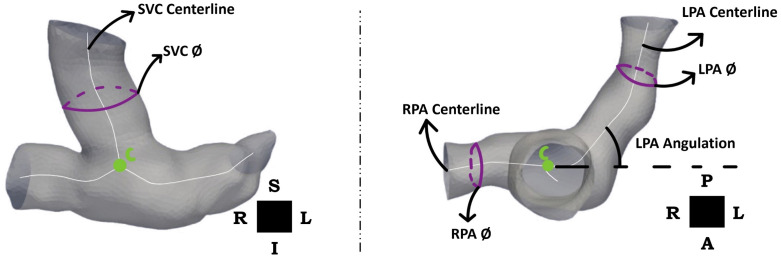
Morphological measurements obtained, represented within one of the 3D geometries.

Conventional morphometric measurements were extracted from the 3D models as shown in Fig 5, and described in Table 1. These measurements were obtained from all patients geometries combining ITK-SNAP [32], VMTK [38] and Paraview [44]. Additionally, volume, surface area and mean diameters were also calculated for the computed SSM template shape.

**Table 1:** Traditional morphological parameters measured on the SCPC 3D models, as shown in Fig 5.

Morphological parameters	Description
3D Volume [mm^3^], Vol	3D volume of the SCPC.
Surface Area [mm^2^], A_surf_	Surface area of the SCPC
LPA Minimum Diameter [mm], D_LPAmin_	Left pulmonary artery minimum diameter.
LPA Mean Diameter [mm], D_LPA_	Left pulmonary artery mean diameter.
RPA Mean Diameter [mm], D_RPA_	Right pulmonary artery mean diameter.
SVC Mean Diameter [mm], D_SVC_	Superior Vena Cava mean diameter.
LPA Tortuosity, T_LPA_	Left Pulmonary Artery mean tortuosity, calculated with VMTK *vmtkcenterlinegeometry* function.
RPA Tortuosity, T_RPA_	Right Pulmonary Artery mean tortuosity, calculated with VMTK *vmtkcenterlinegeometry* function.
SVC Tortuosity, T_SVC_	Superior Vena Cava mean tortuosity, calculated with VMTK *vmtkcenterlinegeometry* function.
LPA Angulation [º], A_LPA_	Anterior angle calculated from the centerline (CL) of the left pulmonary artery (LPA) to the centerline (CL) of the superior vena cava (SVC), viewed from the sagittal plane.
Ratio LPA/RPA mean Diameter, D_LPA_/D_RPA_	Ratio between Left pulmonary artery (LPA) and right pulmonary artery’s mean diameter.
Ratio LPA/SVC mean Diameter, D_LPA_/D_SVC_	Ratio between Left pulmonary artery and Superior vena cava’s diameter.
Ratio LPA Minimum/LPA Mean, D_LPAmin_/ D_LPA_	Ratio between minimum and mean Left pulmonary artery’s diameter.

### Statistical analysis

Mean values and standard deviations (mean±SD) were calculated for volume, surface area and mean diameters of the SCPC. Bivariate correlation analyses were used to investigate relationships between (1) anatomical 3D shape features extracted from SSM and clinical outcomes; and (2) traditional morphological parameters and clinical outcomes. Pearson’s and Spearman’s correlation coefficient (r or ρ) was used to quantify associations when data met parametric assumptions (Pearson) or no-parametric ones (Spearman), which were assessed via the Shapiro–Wilk normality test (significance threshold set at p < 0.05). In cases where normality was not met, non-parametric interpretation was applied. The test was considered statistically significant if the p-value was below 0.05. Cook’s distance was calculated for each model to assess the influence of individual data points and those whose Cook’s distance exceeded twice the mean value were excluded. All statistical analyses were conducted using Python 3.11 (Python Software Foundation, 2024).

Given the relatively small sample size (N = 29) and the high dimensionality of the initial shape data, a comprehensive validation framework was implemented to ensure the reliability of the PLS-derived associations. First, permutation testing with 5,000 shuffles was performed to derive empirical p-values, confirming that the observed correlations were not chance artifacts [45]. Second, a leave-one-out cross-validation (LOO-CV) approach was used to evaluate model generalizability; the degree of ‘shrinkage’ (the difference between the original correlation and the cross-validated correlation) was calculated to assess potential overfitting. Third, bootstrapping (BCa method, 5,000 resamples) was utilized to compute 95% confidence intervals for the effect sizes [46]. Finally, a sensitivity analysis was conducted to determine the statistical power achieved for each clinical outcome given the observed effect sizes.

## Results

### 3D mean shape: Superior cavopulmonary connection complex

**Fig 6:**
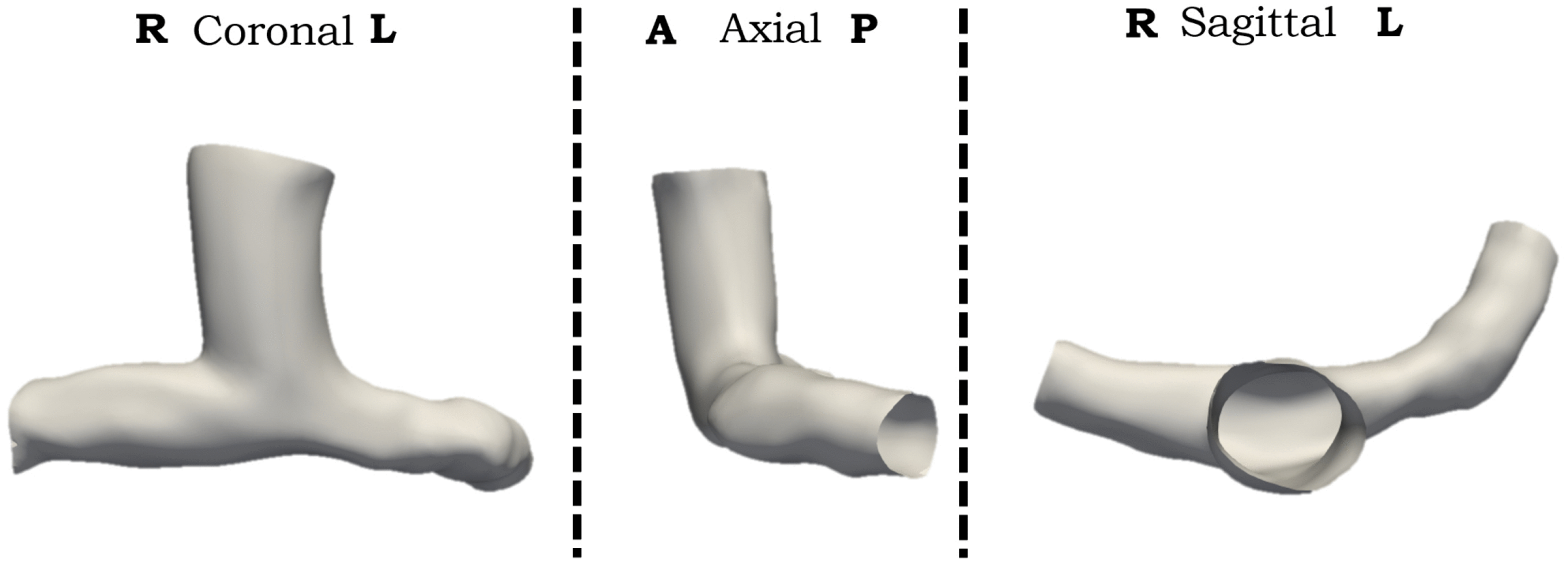
3D template shape of the superior cavopulmonary connection complex computed via statistical shape modelling of 29 patients post bidirectional Glenn procedure.

The 3D mean shape representing the average SCPC “template” shape of the 29 post-bidirectional Glenn patients is shown in Fig 6. The final template captured the distinctive features of Norwood and non-Norwood patients: the template captured narrowing in the LPA and a distinct bulge, corresponding to anatomical features of Norwood and non-Norwood subgroups, respectively. Conventional morphometric parameters extracted from the template closely matched the cohort means (Table 2), showing an overall deviation of 3.4% (Volume: 6.96%, Surface Area: 0.37%, SVC diameter: 0.98%, LPA diameter: 6.06%, RPA diameter: 2.6%). Additionally, cross-validation analyses demonstrated that randomly excluding subjects from the cohort did not significantly alter the template morphology, with an overall surface area deviation of 5.95%.

**Table 2:** Comparison between morphological parameters (3D Volume, Surface area, SVC diameter, LPA diameter and RPA diameter) measured on the 29 3D models of patients (average and standard deviation of the population) and those measured on the SSM computed 3D template.

	3D Vol [mm^3^]	${𝐀}_{𝐬𝐮𝐫𝐟}$[${𝐦𝐦}^{2}$]	D_svc_ [mm]	D_LPA_ [mm]	D_RPA_ [mm]
**Population** (n = 29)	4856 (±1835)	2149 (±520)	10.2 (±1.1)	6.6(±1.5)	7.7 (±1.0)
**Template**	4518	2141	10.3	7.0	7.9

### Relationship between PLS shape features and clinical outcomes

**Fig 7:**
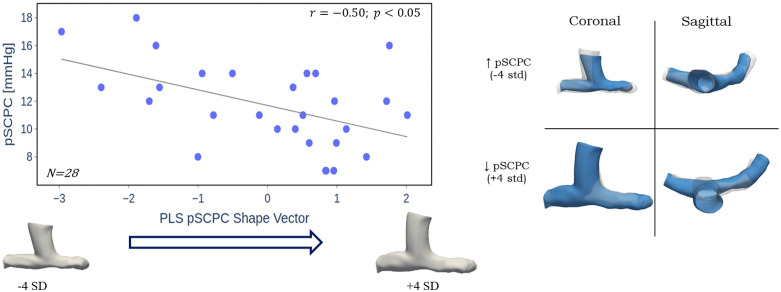
Partial Least Squares analysis of Superior cavopulmonary connection pressure (pSCPC). (Left) PLS shape vector associated with pSCPC from -4 standard deviations (SD) (high pressure) to +4SD (low pressure); (Right) Coronal and sagittal views of computed template shape deformed along the PLS pSCPC shape mode (±4 SD) (in blue) compared to the SSM mean template shape of the population (in light grey).

PLS regression revealed a significant correlation between the PLS shape vector associated with pSCPC pressure and the actual pSCPC pressure values (r = -0.50, p = 0.006) (Fig 7). One patient (P1) was excluded from this analysis due to missing pressure data. Regarding the 3D shape deformation pattern associated with pSCPC pressure, lower pressure values were associated with a straightened, less angulated and slightly larger SVC (corrected for BSA), along with a reduced angulation of the left pulmonary artery compared to cases with higher pSCPC pressure. Furthermore, when conducting the correlation analysis between the pSCPC PLS shape vector and various morphometric parameters, we observed statistically significant associations in several instances (Table 3), corroborating that the PLS shape vector correctly captures a combination of actual morphometric differences between patients. A positive correlation was found with the mean diameter of the LPA. Statistical associations between the pSCPC PLS shape vector and morphometric parameters (LPA diameter, SVC tortuosity, and DLPA/DSVC ratio) are summarized in Table 3. Finally, a significant correlation was also identified between the diameter ratio of the SVC to the LPA. However, when performing the correlations between the morphological parameters and pSCPC, no correlations were observed.

**Table 3:** Correlations between pSCPC clinical parameter and PLS pSCPC shape vector with the previously measured parameters.

Parameter	PLS pSCPC Shape Vector	pSCPC [mmHg]
pSCPC [mmHg]	r=−0.50^**^ (*p*<0.05)	−
iD_LPAmin_ [mm/mm^2^]	ρ =0.21 (*p*=0.29)	ρ =−0.34 (*p*=0.08)
iD_LPAmean_ [mm/mm^2^]	ρ =0.37^*^ (*p*=0.05)	ρ =−0.31 (*p*=0.10)
iD_RPAmean_ [mm/mm^2^]	r=0.44^*^ (*p*<0.05)	r=−0.24 (*p*=0.23)
iD_SVCmean_ [mm/mm^2^]	r=0.14 (*p*=0.47)	r=−0.19 (*p*=0.33)
T_LPA_	ρ=0.32 (*p*=0.10)	ρ =−0.34 (*p*=0.08)
T_RPA_	ρ=−0.21 (*p*=0.28)	ρ =0.20 (*p*=0.30)
T_SVC_	ρ=0.39^*^ (*p*<0.05)	ρ =0.08 (*p*=0.67)
A_LPA_ [º]	ρ=0.18 (*p*=0.37)	ρ =−0.26 (*p*=0.19)
D_LPA_/ D_RPA_	r=−0.07(*p*=0.74)	r=−0.11 (*p*=0.59)
D_LPA_/ D_SVC_	r=0.42^*^ (*p*<0.05)	r=−0.16 (*p*=0.41)
D_LPAmin_/ D_LPAmean_	r=−0.21 (*p*=0.28)	r=−0.17 (*p*=0.39)

**Fig 8:**
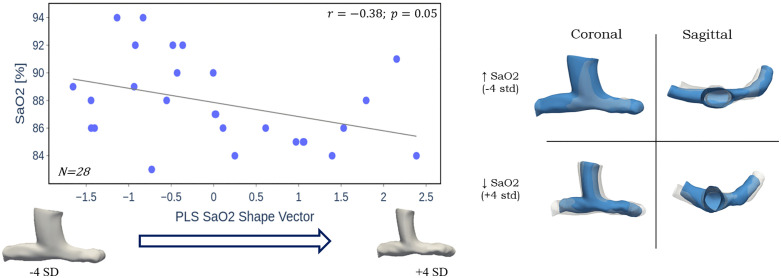
Partial Least Squares analysis of oxygen saturation (SaO_2_). (Left) PLS shape vector associated with pSCPC from -4 standard deviations (SD) (high oxygen) to +4SD (low oxygen); (Right) Coronal and sagittal views of computed template shape deformed along the PLS SaO_2_ shape mode (±4 SD) (in blue) compared to the SSM mean template shape of the population (in light grey).

Regarding the PLS shape vector associated with SaO_2_, no statistically significant correlation was found between the clinical parameter and its corresponding shape vector (r = -0.382, p = 0.052), as shown in Fig 8. One patient (P1) was excluded due to missing clinical data, and an additional subject (P29) was removed as it was considered an outlier based on the Cook’s distance analysis. We did find several features strongly correlated with the SaO_2_ PLS Shape vector such as: LPA related measurements, including tortuosity and the ratio between the minimum and mean diameters Table 4. Moreover, Fig 8 illustrates lateral elongation of the vascular complex at higher SaO_2_ levels. Conversely, lateral narrowing is observed in cases of lower SaO_2_ levels. However, in this case, no correlations were found between SaO_2_ and the other measured morphological parameters.

**Table 4:** Correlations between oxygen saturation (SaO_2_) clinical parameter and PLS SaO_2_ shape vector with the previously measured parameters.

Parameter	PLS SaO_2_ Shape Vector	SaO_2_ [%]
SaO_2_ [%]	r=−0.38^*^ (*p*=0.05)	−
iD_LPAmin_ [mm/mm^2^]	ρ =0.58^**^ (*p*<0.05)	ρ=−0.21 (*p*=0.29)
iD_LPAmean_ [mm/mm^2^]	ρ =0.59^**^ (*p*<0.05)	ρ=−0.31 (*p*=0.11)
iD_RPAmean_ [mm/mm^2^]	r=0.08 (*p*=0.70)	r=0.21 (*p*=0.29)
iD_SVCmean_ [mm/mm^2^]	r=0.23 (*p*=.243)	r=−0.13 (*p*=0.52)
T_LPA_	ρ =0.51^**^ (*p*<0.05)	ρ=−0.11 (*p*=0.61)
T_RPA_	ρ=0.00 (*p*=0.99)	ρ=−0.11 (*p*=0.59)
T_SVC_	ρ=−0.09 (*p*=0.63)	ρ=0.14 (*p*=0.48)
A_LPA_ [º]	ρ=0.32 (*p*=0.10)	ρ=0.05 (*p*=0.82)
D_LPA_/ D_RPA_	r=−0.49^**^(*p*<0.05)	r=−0.24 (*p*=0.23)
**D**_**LPA**_ **/ D**_**SVC**_	r=−0.05 (*p*=0.79)	r=−0.15 (*p*=0.47)
**D**_**LPAmin**_ **/ D**_**LPAmean**_	r=0.52^**^ (*p*<0.05)	r=−0.14 (*p*=0.49)

**Fig 9:**
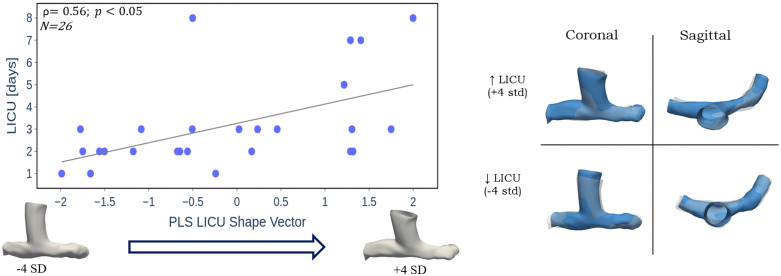
Partial Least Squares analysis of the length of ICU stay (LICU). (Left) PLS shape vector associated with LICU from -4 standard deviations (SD) (few days in ICU) to +4SD (many days in ICU); (Right) Coronal and sagittal views of computed template shape deformed along the PLS LICU shape mode (±4 SD) (in blue) compared to the SSM mean template shape of the population (in light grey).

For the length of stay in the ICU (LICU), a significant correlation was found between the clinical parameter and its corresponding PLS shape vector (ρ = 0.56, ρ < 0.05) as shown in Fig 9. Three patients (P29, P13, and P3) were excluded from this analysis due to missing LICU data. The 3D shape representation (Fig 9) demonstrates a pronounced narrowing of the LPA in patients who spent more days in the LICU. Specifically, we found correlations between this clinical parameter and morphological parameters such as: the mean diameter of the LPA, the ratio between the mean diameters of the LPA and RPA, and finally, the ratio between the minimum and mean diameters of the LPA (Table 5).

**Table 5:** Correlations between the length of ICU stay (LICU) clinical parameter and PLS LICU shape vector with the previously measured parameters.

Parameter	PLS LICU Shape Vector	LICU [days]
LICU [days]	ρ=0.56^**^ (*p*=0.00)	−
iD_LPAmin_ [mm/mm^2^]	r=−0.64^***^ (*p*<0.05)	ρ=−0.31 (*p*=0.13)
iD_LPAmean_ [mm/mm^2^]	r=−0.54^**^ (*p*<0.05)	ρ=−0.45^*^ *p*<0.05)
iD_RPAmean_ [mm/mm^2^]	r=−0.06 (*p*=0.78)	ρ=0.26 (*p*=0.20)
iD_SVCmean_ [mm/mm^2^]	r=−0.09 (*p*=0.66)	ρ=0.06 (*p*=0.79)
T_LPA_	ρ=−0.35 (*p*=0.49)	ρ=−0.33 (*p*=0.11)
T_RPA_	ρ=−0.03 (*p*=0.87)	ρ=−0.38 (*p*=0.06)
T_SVC_	ρ=−0.14 (*p*=0.09)	ρ=−0.27 (*p*=0.18)
A_LPA_ [º]	ρ =−0.17 (*p*=0.42)	ρ=−0.11 (*p*=0.58)
D_LPA_/ D_RPA_	r=−0.43^*^(*p*<0.05)	ρ=−0.57^**^ (*p*<0.05)
D_LPA_/ D_SVC_	r=−0.00 (*p*=0.99)	ρ=0.21 (*p*=0.30)
D_LPAmin_/ D_LPAmean_	r=−0.58^**^ (*p*<0.05)	ρ=−0.49^*^ (*p*<0.05)

**Fig 10:**
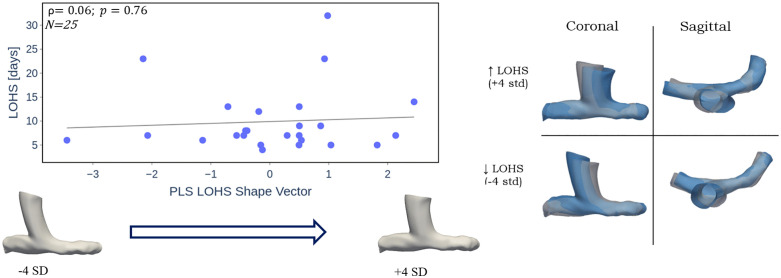
Partial Least Squares analysis of length of hospital stay (LOHS). (Left) PLS shape vector associated with LOHS from -4 standard deviations (SD) (few days in hospital) to +4SD (many days in hospital). (Right) Coronal and sagittal views of computed template shape deformed along the PLS LOHS shape mode (±4 SD) (in blue) compared to the SSM mean template shape of the population (in light grey).

Finally, no significant correlation was found between the number of days spent in the hospital and its corresponding PLS shape vector (ρ = 0.063, ρ = 0.76). The same three patients excluded in the LICU analysis (P29, P13, and P3) were also removed here due to missing data, along with an additional subject (P27) excluded based on the Cook’s distance analysis. Fig 10 illustrates shape variations in SVC orientation and LPA angulation associated with LOHS, although these did not reach statistical significance. Regarding the observed correlations, we found a statistically significant association between the LOHS PLS shape vector and the diameter of the superior vena cava (SVC), which is further supported by the direct correlation between the length of hospital stay and the SVC diameter (Table 6).

**Table 6:** Correlations between length of hospital stay (LOHS) clinical parameter and PLS LOHS shape vector with the previously measured parameters.

Parameter	PLS LOHS Shape Vector	LOHS [days]
LOHS [days]	ρ=0.06 (*p*=0.76)	−
iD_LPAmin_ [mm/mm^2^]	r=−0.02 (*p*=0.92)	ρ=−0.10 (*p*=0.62)
iD_LPAmean_ [mm/mm^2^]	r=−0.14 (*p*=0.50)	ρ=−0.37 (*p*=0.07)
iD_RPAmean_ [mm/mm^2^]	r=−0.08 (*p*=0.70)	ρ=−0.18 (*p*=0.39)
iD_SVCmean_ [mm/mm^2^]	r=−0.42^*^ (*p*<0.05)	ρ=−0.48^*^ (*p*<0.05)
T_LPA_	ρ=−0.02 (*p*=0.93)	ρ=−0.15 (*p*=0.45)
T_RPA_	ρ=0.17 (*p*=0.41)	ρ=−0.13 (*p*=0.52)
T_SVC_	ρ=−0.02 (*p*=0.92)	ρ=−0.28 (*p*=0.16)
A_LPA_ [º]	ρ=−0.17 (*p*=0.42)	ρ=−0.15 (*p*=0.47)
D_LPA_/ D_RPA_	r=−0.09 (*p*=0.67)	ρ=−0.21 (*p*=0.30)
D_LPA_/ D_SVC_	r=0.16 (*p*=0.46)	ρ=0.10 (*p*=0.62)
D_LPAmin_/ D_LPAmean_	r=0.02 (*p*=0.94)	ρ=−0.16 (*p*=0.45)

To further corroborate our findings and address potential concerns regarding the sample size (N = 29) and the risk of overfitting, additional robust statistical tests were conducted as part of a comprehensive validation framework. The PLS analysis revealed significant associations between 3D morphology and the primary clinical outcomes (Table 7). For pSCPC pressure, a strong negative correlation was observed (r = -0.50, p_perm_ = 0.007), demonstrating high stability in cross-validation (shrinkage = 0.133) and a statistical power of 83%, with the 95% bootstrap confidence interval (BCa CI: [-0.73, -0.15]) not crossing zero. Similarly, LICU stay showed a significant positive correlation (ρ= + 0.56, p_perm_ = 0.005), with a shrinkage of 0.139 and a statistical power of 84% (BCa CI: [+0.23, + 0.78]).

**Table 7:** Summary results for validation tests based on the correlations between the PLS shape vector of each clinical parameter and its corresponding clinical parameter.

Outcome	p_perm_	Shrinkage	Jackknife ± std	CI 95% (BCa)	Achieved power
**pSCPC**	0.007	0.133	−0.50 ± 0.03	[−0.731, −0.146]	83%
**SaO2**	0.051	0.191	−0.38 ± 0.03	[−0.663, +0.002]	51%
**LICU**	0.005	0.139	+0.56 ± 0.03	[+0.225, +0.780]	84%
**LOHS**	0.767	−0.490	+0.06 ± 0.05	[−0.341, +0.447]	6%

In contrast, associations for SaO_2_ (r = -0.38, p_perm_ = 0.051, power = 51%) and LOHS (ρ = 0.06, p_perm_ = 0.767, power = 6%) were less conclusive under these rigorous validation tests, suggesting that these variables may be more influenced by non-geometric clinical factors, as further detailed in the Discussion.

## Discussion

The bidirectional Glenn procedure is a key intermediate stage in the surgical management of patients with single ventricle physiology, such as those with tricuspid atresia or hypoplastic left heart syndrome. Despite advances in surgical technique and perioperative care, mortality and morbidity between the Glenn and Fontan stages have remained largely unchanged, highlighting the need for improved risk stratification tools and a better understanding of the factors influencing patient outcomes during this critical period [47,48].

In this study, thanks to the 3D tools employed by the SSM, we have been able to first generate an average, “prototype” or template shape of the SCPC and extract the predominant morphological features present in patients with single ventricle (SV) physiology based on various clinical parameters. This method, which combines CMR-based computational technology with advanced statistical analysis, relies on the definition of a template derived from the 3D geometries of the patients within our cohort.

The template constructed from the geometries of 29 patients represents the average shape across all subjects and effectively captures the morphological differences observed in the LPA among them. As previously described, some patients exhibit a mild stenosis along the artery, while others display a bulging region. The template successfully integrates both variations and reflects them in the final 3D prototype (Fig 6). Furthermore, the surface registration and alignment process was remarkably accurate, despite the observed anatomical differences in the LPA among patients (Fig 3). Due to the small number of patients included in the study, we observed greater variability in vascular shape. Increasing the sample size could help to more accurately define the average morphology in this patient population. Nevertheless, even with a limited cohort, we identified morphological patterns characteristic of single ventricle (SV) patients. These patterns are consistent with findings reported in numerous studies [49–51], which often highlight a narrowing of the left pulmonary artery (LPA). In our resulting template, rather than observing a localized stenosis, we identified a diffuse narrowing along the entire length of the LPA. This observation could be further explored through, for example, computational fluid dynamics (CFD) or 4D flow studies to assess the extent to which this narrowing might negatively impact future hemodynamic performance in these patients.

One of the most prominent morphological features identified in SV patients is stenosis of the LPA. In many such cases, treatment ultimately involves patch augmentation to improve pulmonary arterial physiology [52]. While no focal stenosis had been clinically identified in the studied population, results suggest that even a general narrowing of the LPA (as shown in Fig 6) may be associated with adverse short-term outcomes. Using this template, and with the assistance of Partial Least Squares (PLS) analysis, we were able to construct shape vectors based on different clinical parameters.

We identified distinct 3D shape features associated with adverse configurations of the clinical parameters studied. For high pSCPC values, we observed a more flattened LPA morphology, while low pSCPC values corresponded to an elongated and unfolded LPA configuration. The statistical shape analysis revealed a significant correlation between the 3D shape vector and pSCPC, suggesting that a compact, flattened LPA geometry may contribute to higher pressures in the SCPC complex. Although direct correlations between pSCPC and individual morphometric descriptors (e.g., diameters) were not statistically significant, our results suggest a potential trend—namely, that larger diameters could be associated with a more open and elongated LPA, potentially reducing pressure. A larger sample size would be needed to confirm this hypothesis. Identifying these morphological biomarkers at the pre-Fontan stage has direct clinical implications for surgical planning. For instance, the association between a flattened, compact LPA morphology and higher pSCPC suggests that patients exhibiting this phenotype may require more targeted surgical reconstruction—such as extensive patch augmentation—during the Fontan procedure to lower pulmonary resistance. By proactively identifying suboptimal geometries through SSM, surgeons could tailor the Fontan completion to mitigate the risk of early hemodynamic failure.

Regarding SaO_2_, we did not find a clear correlation, although the results were close to statistical significance. In this case, despite the lack of statistical significance, the 3D shape suggests a larger SCPC complex and a flattening in the anterior-posterior direction to be more strongly associated with higher SaO_2_ values. Furthermore, we observed correlations with most LPA-related morphological parameters. This indicates that diameter and tortuosity may play a role in oxygen saturation. However, as with the previous case, no significant correlations were found between the clinical parameter and the respective morphological descriptors. Given these findings, it would be beneficial to increase the sample size in order to confirm statistical significance.

With respect to ICU stay, we found correlations between all LPA diameter-related morphological parameters. All correlations were negative, indicating that smaller diameters may be associated with longer ICU stays. In the 3D models, longer ICU durations appeared to coincide with stenosis along the LPA. In addition, we observed correlations between ICU days and some specific morphological parameters, such as mean LPA diameter and RPA-to-LPA diameter ratios, which support the findings obtained from the morphometric analysis and the PLS shape vector.

For length of hospital stay (LOHS) following the Glenn procedure, we did not identify a clear pattern in the 3D shape vector that correlates with this parameter, possibly due to confounding external factors such as infection, social context, or family preferences. Nevertheless, the obtained results may suggest that a smaller SVC may be linked to prolonged hospitalization. While this result is promising, additional data are needed to validate this association given the multifactorial nature of LOHS. Furthermore, the morphological features identified as adverse (e.g., diffuse LPA narrowing and increased SVC angulation) may serve as early indicators of long-term morbidity. A suboptimal Glenn geometry that correlates with prolonged ICU stays or borderline pressures could predispose the patient to chronic complications after Fontan completion, such as protein-losing enteropathy or decreased exercise capacity, which are often driven by inefficient pulmonary hemodynamics. Using SSM-derived shape scores as a risk-stratification tool could therefore help identify a subset of ‘high-risk’ patients who require closer longitudinal monitoring or early catheter-based interventions to optimize pulmonary arterial growth before the circulation is fully transitioned to the Fontan stage.

In summary, we developed a population-based average SCPC template derived from SV patients, which provides a realistic morphological baseline that could support post-Glenn and pre-Fontan assessment or risk stratification. Unlike simplified representations—such as idealized “T-shaped” geometries—this template reflects anatomical characteristics observed in actual patients, and thus may offer more clinically relevant input for preoperative simulations. Additionally, this population-derived model could be used in combination with PCA-based shape variation to generate a wide spectrum of valid SCPC geometries, enabling simulation of a broad range of hemodynamic scenarios. Beyond the creation of this template, this study allowed us, for the first time, to describe predominant morphological features associated with SCPC pressure, SaO_2_ levels, and ICU length of stay. These findings provide valuable insights that could help reduce the still high mortality and morbidity rates prior to the Fontan operation. In the future, developing separate templates for specific underlying pathologies or surgical subtypes may offer a more granular understanding of morphological variability across subgroups. Increasing the number of patients would also help reduce shape variability and reveal additional morphometric features currently undetectable in this limited cohort. Furthermore, using the distinct PLS vectors derived for each clinical parameter, CFD or 4D Flow studies could be conducted to explore how hemodynamics vary with specific shape deformations.

### Limitations

The conclusions of our study are based on a cohort of patients retrospectively recruited from a single center, which might not be fully representative of the broader population of congenital single ventricle patients undergoing the Bidirectional Glenn procedure. While the sample size (n = 29) is comparable to other pilot studies applying statistical shape modeling in this rare disease context [53–56], it remains relatively small. This may limit the statistical power to detect subtle morphological associations or rare anatomical phenotypes. A wider multi-center study would be necessary to provide additional insights and validate the generalizability of our findings.

In addition to this, our study’s scope was restricted to examining the relationship between post-operative 3D anatomy and early-to-mid-term clinical outcomes (e.g., ICU stay and SCPC pressure). A significant limitation in this regard is the absence of longitudinal 3D data to account for preoperative anatomical and hemodynamic factors. In our clinical practice at GOSH, high-resolution 3D CMR is typically performed at the pre-Fontan stage and is not routinely acquired for neonates prior to the first surgical stage. Consequently, our findings describe the post-operative morphological phenotype and its association with clinical outcomes at a specific clinical milestone, without making inferences about the specific effects of surgical remodeling or the impact of preoperative developmental changes.

While these are validated clinical indicators, they do not fully characterize the long-term hemodynamic consequences or the transition to the Fontan circulation. A future step would be to gather longitudinal data regarding the development of aortopulmonary collaterals, pulmonary artery growth over time, and exercise capacity. Moreover, the assessment of SCPC pressure and oxygen saturation was retrospective and, despite being collected based on standardized clinical guidelines, inter-operator and inter-batch variability in catheterization and monitoring could be present.

The Statistical Shape Modeling (SSM) framework has its own technical limitations. We primarily considered linear relationships between shape vectors and clinical variables via PLS regression; therefore, higher-order or non-linear interactions might have been underestimated. Furthermore, the representation of the SCPC morphology is inherently limited by the segmentation process. The registration process was rigorously validated using Principal Component Analysis (PCA). We confirmed that the first principal components captured true morphological variance rather than artifacts related to patient orientation. The absence of rotational components in the primary modes of variation demonstrates that all subjects were consistently and correctly aligned within the common coordinate system. All data were acquired and processed at Great Ormond Street Hospital (GOSH), specialized centre in the management of complex congenital heart disease. The clinical environment ensures that all imaging protocols follow the highest pediatric cardiac standards. The image segmentation workflow involved a rigorous collaborative review process. Following initial segmentation, each 3D model was individually inspected and verified by a multidisciplinary team composed of senior cardiovascular radiologists and bioengineers. This step ensures that the resulting 3D geometries are anatomically faithful and clinically validated before undergoing Statistical Shape Modelling (SSM).

Finally, the relationship between 3D shape and clinical outcomes was simplified by focusing on global morphological deformations. This approach does not explicitly account for the impact of non-geometric factors, such as individual pulmonary vascular resistance (PVR) or the presence of sub-clinical microscopic changes in the pulmonary vasculature. While our SSM framework successfully identified key morphological biomarkers, modeling the interaction between 3D geometry, computational fluid dynamics (CFD), and patient-specific biological factors would be a natural and necessary extension of this work.

## Supporting information

S1 Appendix Clinical data extracted from 29 single ventricle (SV) patients. (DOCX)

## Abbreviations

ASD: Atrial septal defect
CMR: Cardiovascular Magnetic Resonance
DILV: Double inlet left ventricle
DORV: Double outlet right ventricle
HLHS: Hypoplastic Left Heart syndrome
IVC: Inferior Vena Cava
LPA: Left Pulmonary artery
MA: Mitral Atresia
PA/IVS: Pulmonary atresia with intact ventricular septum
PLS: Partial Least Square
SCPC: Superior Cavopulmonary connection
SSM: Statistical Shape Modelling
SV: Single Ventricle
SVC: Superior Vena Cava
TA: Tricuspid atresia
TGA: Transposition of the great arteries
VSD: Ventricular septal defect

## References

[1] Lynn MM, Salemi JL, Kostelyna SP, Morris SA, Sexson Tejtel SK, Lopez KN. Lesion-specific congenital heart disease mortality trends in children: 1999 to 2017. Pediatrics. 2022;150(4):2022056294. doi: 10.1542/peds.2022-056294 36047307 PMC9645438

[2] Salari N, Faryadras F, Shohaimi S, Jalili F, Hasheminezhad R, Babajani F, et al. Global prevalence of congenital heart diseases in infants: a systematic review and meta-analysis. J Neonatal Nurs. 2024;30(6):570–5. doi: 10.1016/j.jnn.2024.05.003

[3] Kong ASY, Khaw KY, Mood MC, Khan MA, Ayub Q, Maran S. The burden of congenital heart disease in Malaysia: a comprehensive review and meta-analytic synthesis, 1970–2024. BMC Public Health. 2025;25(1):2595. doi: 10.1186/s12889-025-23800-2 40739499 PMC12308901

[4] Opotowsky AR, Khairy P, Diller G, Kasparian NA, Brophy J, Jenkins K, et al. Clinical Risk Assessment and Prediction in Congenital Heart Disease Across the Lifespan: JACC Scientific Statement. J Am Coll Cardiol. 2024;83(21):2092–111. doi: 10.1016/j.jacc.2024.02.055 38777512

[5] Mohsin SN, Gapizov A, Ekhator C, Ain NU, Ahmad S, Khan M, et al. The role of artificial intelligence in prediction, risk stratification, and personalized treatment planning for congenital heart diseases. Cureus. 2023;15(8):e44374. doi: 10.7759/cureus.44374 37664359 PMC10469091

[6] Rao PS. Single ventricle—a comprehensive review. Children. 2021;8(6):441. doi: 10.3390/children8060441 34073809 PMC8225092

[7] Mastropietro CW, Clark JA, Grimaldi LM, Killinger JS, Richmond M. The patient with a single cardiac ventricle. Curr Pediatr Rev. 2012;8(3):253–76. doi: 10.2174/157339612802139442

[8] Mayer JE Jr, Bridges ND, Lock JE, Hanley FL, Jonas RA, Castaneda AR. Factors associated with marked reduction in mortality for Fontan operations in patients with single ventricle. J Thorac Cardiovasc Surg. 1992;103(3):444–51; discussion 451-2. doi: 10.1016/s0022-5223(19)34983-9 1545543

[9] Weber RW, Stiasny B, Ruecker B, Fasnacht M, Cavigelli-Brunner A, Valsangiacomo Buechel ER. Prenatal diagnosis of single ventricle physiology impacts on cardiac morbidity and mortality. Pediatr Cardiol. 2019;40(1):61–70. doi: 10.1007/s00246-018-1961-1 30121866

[10] Vodiskar J, Kido T, Strbad M, Cleuziou J, Hager A, Ewert P. Outcomes of single ventricle palliation in infants with heterotaxy syndrome. Eur J Cardiothorac Surg. 2021;60(3):554–61. doi: 10.1093/ejcts/ezab141 33783481

[11] Corno AF, Findley TO, Salazar JD. Narrative review of single ventricle: where are we after 40 years? Transl Pediatr. 2023;12(2):221–44. doi: 10.21037/TP-22-573 36891374 PMC9986776

[12] Juaneda I, Chiostri B, Kreutzer C. Technical aspects and common pitfalls of norwood with modified Blalock Taussig Shunt. World J Pediatr Congenit Heart Surg. 2022;13(5):576–80. doi: 10.1177/21501351221111798 36053104

[13] Azzolina G, Eufrate S, Pensa P. Tricuspid atresia: experience in surgical management with a modified cavopulmonary anastomosis. Thorax. 1972;27(1):111–5. doi: 10.1136/THX.27.1.111 5017561 PMC472475

[14] Bucholz EM, Lu M, Sleeper L, Vergales J, Bingler MA, Ronai C, et al. Risk factors for death or transplant after stage 2 palliation for single ventricle heart disease. JACC Adv. 2024;3(5):100934. doi: 10.1016/J.JACADV.2024.100934 38939642 PMC11198479

[15] Arrigoni SC, Ebels T. Bilateral bidirectional cavopulmonary connection: a review of surgical techniques and clinical implications. Transl Pediatr. 2024;13(5):814–23. doi: 10.21037/tp-24-28 38840681 PMC11148731

[16] Javadi E, Laudenschlager S, Kheyfets V, Di Maria M, Stone M, Jamali S, et al. Predicting hemodynamic performance of Fontan operation for Glenn physiology using computational fluid dynamics: ten patient-specific cases. J Clin Images Med Case Rep. 2022;3(6):1916. doi: 10.52768/2766-7820/1916 36339935 PMC9631545

[17] Kogon BE, Plattner C, Leong T, Simsic J, Kirshbom PM, Kanter KR. Outcomes After Bidirectional Glenn Shunt in a Tertiary-Care Pediatric Hospital in South Africa. J Cardiothorac Vasc Anesth. 2022;36(6):1573–81. doi: 10.1016/j.jtcvs.2008.05.017 35151565

[18] Tariq M, Zahid I, Hashmi S, Amanullah M, Shahabuddin S. The Glenn procedure: Clinical outcomes in patients with congenital heart disease in Pakistan. Ann Card Anaesth. 2021;24(1):30–5. doi: 10.4103/aca.ACA_85_19 33938828 PMC8081130

[19] Greenberg JW, Pribble CM, Singareddy A, Ta N-A, Sescleifer AM, Fiore AC, et al. The failed bidirectional Glenn shunt: risk factors for poor outcomes and the role of early reoperation. World J Pediatr Congenit Heart Surg. 2021;12(6):760–4. doi: 10.1177/21501351211044129 34846973

[20] Abdelmohsen GA, Gabel HA, Alamri RM, Baamer A, Al-Radi OO, Binyamin A, et al. Bidirectional glenn surgery without palliative pulmonary artery banding in univentricular heart with unrestricted pulmonary flow. Retrospective multicenter experience. J Cardiothorac Surg. 2024;19(1):67. doi: 10.1186/s13019-024-02572-7 38321557 PMC10845678

[21] Ghelani SJ, Beroukhim R, Thatte N. CMR for planning staged biventricular repair in children with complex congenital heart diseases. J Cardiovasc Magn Reson. 2025;27:101817. doi: 10.1016/j.jocmr.2024.101817

[22] Stern KWD, McElhinney DB, Gauvreau K, Geva T, Brown DW. Echocardiographic evaluation before bidirectional Glenn operation in functional single-ventricle heart disease: comparison to catheter angiography. Circ Cardiovasc Imaging. 2011;4(5):498–505. doi: 10.1161/CIRCIMAGING.110.963280 21730025

[23] Swanson L, Sauvage E, Ngoepe M, Schievano S, Bruse JL, Hsia T-Y. The enduring impact of shape following perfect coarctation of the aorta repair. World J Pediatr Congenit Heart Surg. 2025;16(1):30–6. doi: 10.1177/21501351241269868 39285811

[24] Williams JG, Marlevi D, Bruse JL, Nezami FR, Moradi H, Fortunato RN, et al. Aortic dissection is determined by specific shape and hemodynamic interactions. Ann Biomed Eng. 2022;50(12):1771–86. doi: 10.1007/S10439-022-02979-0 35943618 PMC11262626

[25] Van Santvliet L, Zappon E, Gsell MAF, Thaler F, Blondeel M, Dymarkowski S, et al. Integrating anatomy and electrophysiology in the healthy human heart: Insights from biventricular statistical shape analysis using universal coordinates. Comput Biol Med. 2025;192(Pt A):110230. doi: 10.1016/j.compbiomed.2025.110230 40324309

[26] Sophocleous F, Bône A, Shearn AIU, Forte MNV, Bruse JL, Caputo M, et al. Feasibility of a longitudinal statistical atlas model to study aortic growth in congenital heart disease. Comput Biol Med. 2022;144:105326. doi: 10.1016/j.compbiomed.2022.105326 35245697

[27] Bruse JL, Khushnood A, McLeod K, Biglino G, Sermesant M, Pennec X, et al. How successful is successful? Aortic arch shape after successful aortic coarctation repair correlates with left ventricular function. J Thorac Cardiovasc Surg. 2017;153(2):418–27. doi: 10.1016/j.jtcvs.2016.09.018 27776913

[28] Azimi M, de Almeida DF, Khamooshi M, Liao S, Šeman M, Taylor A, et al. Statistical shape modelling of the left ventricle for patients with HeartMate2 and HeartMate3 ventricular assist devices. Comput Biol Med. 2025;189:109921. doi: 10.1016/j.compbiomed.2025.109921 40031108

[29] Bruse JL, McLeod K, Biglino G, Ntsinjana HN, Capelli C, Hsia T-Y, et al. A statistical shape modelling framework to extract 3D shape biomarkers from medical imaging data: assessing arch morphology of repaired coarctation of the aorta. BMC Med Imaging. 2016;16(1):40. doi: 10.1186/s12880-016-0142-z 27245048 PMC4894556

[30] Rodriguez-Florez N, Bruse JL, Borghi A, Vercruysse H, Ong J, James G, et al. Statistical shape modelling to aid surgical planning: associations between surgical parameters and head shapes following spring-assisted cranioplasty. Int J Comput Assist Radiol Surg. 2017;12(10):1739–49. doi: 10.1007/s11548-017-1614-5 28550406 PMC5608871

[31] Haycock GB, Schwartz GJ, Wisotsky DH. Geometric method for measuring body surface area: a height-weight formula validated in infants, children, and adults. J Pediatr. 1978;93(1):62–6. doi: 10.1016/S0022-3476(78)80601-5 650346

[32] Yushkevich PA, Piven J, Hazlett HC, Smith RG, Ho S, Gee JC, et al. ITK-SNAP [Internet]. Philadelphia, PA, USA: University of Pennsylvania; 2006. Available from: http://www.itksnap.org

[33] Yushkevich PA, Piven J, Hazlett HC, Smith RG, Ho S, Gee JC, et al. User-guided 3D active contour segmentation of anatomical structures: significantly improved efficiency and reliability. Neuroimage. 2006;31(3):1116–28. doi: 10.1016/j.neuroimage.2006.01.015 16545965

[34] Boumpouli M, Sauvage EL, Capelli C, Schievano S, Kazakidi A. Characterization of flow dynamics in the pulmonary bifurcation of patients with repaired tetralogy of fallot: a computational approach. Front Cardiovasc Med. 2021;8:703717. doi: 10.3389/fcvm.2021.703717 34660711 PMC8514754

[35] Sivera R, Clark AE, Dall’Asta A, Ghi T, Schievano S, Lees CC. Fetal face shape analysis from prenatal 3D ultrasound images. Sci Rep. 2024;14(1):1–15. doi: 10.1038/s41598-023-50386-9 38388522 PMC10884000

[36] Autodesk Inc. Meshmixer. San Rafael, CA, USA: Autodesk Inc.; 2025.

[37] Piccinelli M, Veneziani A, Steinman DA, Remuzzi A, Antiga L. A framework for geometric analysis of vascular structures: application to cerebral aneurysms. IEEE Trans Med Imaging. 2009;28(8):1141–55. doi: 10.1109/TMI.2009.2021652 19447701

[38] Izzo R, Steinman D, Manini S, Antiga L. The Vascular Modeling Toolkit: A Python Library for the Analysis of Tubular Structures in Medical Images. J Open Source Softw. 2018;3(25):745. doi: 10.21105/joss.00745

[39] Bône A, Louis M, Martin B, Durrleman S. Deformetrica 4: An Open-Source Software for Statistical Shape Analysis. Lecture Notes in Computer Science. 2018. pp. 3–13. doi: 10.1007/978-3-030-04747-4_1

[40] Vaillant M, Glaunès J. Surface matching via currents. Lectures Notes in Computer Science. 2005. pp. 381–92. doi: 10.1007/11505730_32 17354711

[41] Goparaju A, Iyer K, Bône A, Hu N, Henninger HB, Anderson AE, et al. Benchmarking off-the-shelf statistical shape modeling tools in clinical applications. Med Image Anal. 2022;76:102271. doi: 10.1016/j.media.2021.102271 34974213 PMC8792348

[42] Durrleman S, Prastawa M, Charon N, Korenberg JR, Joshi S, Gerig G, et al. Morphometry of anatomical shape complexes with dense deformations and sparse parameters. Neuroimage. 2014;101:35–49. doi: 10.1016/j.neuroimage.2014.06.043 24973601 PMC4871626

[43] Vrieze SI. Model selection and psychological theory: a discussion of the differences between the Akaike information criterion (AIC) and the Bayesian information criterion (BIC). Psychol Methods. 2012;17(2):228–43. doi: 10.1037/a0027127 22309957 PMC3366160

[44] Kitware Inc. Paraview. Kitware; 2024.

[45] Phipson B, Smyth GK. Permutation p-values should never be zero: calculating exact p-values when permutations are randomly drawn. Stat Appl Genet Mol Biol. 2016;9(1). doi: 10.2202/1544-6115.1585 21044043

[46] Cox D, Hinkley D, Reid N, Rubin D, Silverman B. An Introduction to the Bootstrap. 1994;21(2):32. doi: 10.1201/9780429246593

[47] Weinerman B, Cheung E, Park S. Risk factors and post-operative management for the bidirectional Glenn: a literature review. Curr Pediatr Rep. 2023;11(4):174–9. doi: 10.1007/S40124-023-00294-3

[48] Kobayashi RL, Williams RJ, Gauvreau K, Daly KP, Esteso P, Milligan C, et al. Improving mechanical circulatory support outcomes in failing bidirectional Glenn physiology. Pediatr Cardiol. 2025;46(6):1719–24. doi: 10.1007/s00246-024-03597-4 39030349

[49] Carrillo SA, Best C, Hersey D, Texter K, McConnell PI, Boe B, et al. Preemptive stenting of the left pulmonary artery during comprehensive stage 2 procedure does not influence Fontan candidacy. JTCVS Open. 2022;13:330–43. doi: 10.1016/j.xjon.2022.11.007 37063164 PMC10091295

[50] Rahkonen O, Chaturvedi RR, Benson L, Honjo O, Caldarone CA, Lee K-J. Pulmonary artery stenosis in hybrid single-ventricle palliation: High incidence of left pulmonary artery intervention. J Thorac Cardiovasc Surg. 2015;149(4):1102–10.e2. doi: 10.1016/j.jtcvs.2014.11.080 25595374

[51] Dasi LP, Sundareswaran KS, Sherwin C, de Zelicourt D, Kanter K, Fogel MA, et al. Larger aortic reconstruction corresponds to diminished left pulmonary artery size in patients with single-ventricle physiology. J Thorac Cardiovasc Surg. 2010;139(3):557–61. doi: 10.1016/j.jtcvs.2009.08.023 19880146 PMC2827635

[52] Seaman CS, d’Udekem Y, Jones BO, Brizard CPR, Cheung MMH. Augmentation of Pulmonary Arterial Growth in Single Ventricle Patients by Interim Selective Shunts. Semin Thorac Cardiovasc Surg. 2021;33(2):483–9. doi: 10.1053/j.semtcvs.2020.09.007 32977010

[53] Aljassam Y, Sophocleous F, Bruse J, Schot V, Caputo M, Biglino G. Assessing aortic morphology post aortic valve replacement using unsupervised hierarchical clustering and statistical shape modelling. Eur Heart J. 2024;45(Supplement_1). doi: 10.1093/eurheartj/ehae666.3629

[54] Peymani A, Dobbe I, van Rijn S, McCarroll HR, Streekstra G, Strackee S. P141. A 3D Statistical Shape Model of the Distal Radius In Madelung Deformity. Plast Reconstr Surg Glob Open. 2022;10(4S):116–7. doi: 10.1097/01.gox.0000828904.09810.af

[55] Johnson LG, Mozingo JD, Atkins PR, Schwab S, Morris A, Elhabian S, et al. A three-dimensional statistical shape model to describe clinical shape variation of the proximal femur in patients with Legg-calvé-perthes disease deformity. Osteoarthritis Imaging. 2023;3:100142. doi: 10.1016/j.ostima.2023.100142

[56] Blackman J, Giles JW. A first-of-its-kind two-body statistical shape model of the arthropathic shoulder: enhancing biomechanics and surgical planning. J Orthop Surg Res. 2025;20(1):563. doi: 10.1186/s13018-025-05855-4 40462104 PMC12131784

