# Supplementary material for: Linking shape and clinical outcomes: Statistical shape model of Bidirectional Glenn connection in congenital single ventricle patients
Source: PLOS Digit Health. 2026 Aug 18;5(8):e0001611. doi: 10.1371/journal.pdig.0001611 (PMC13485067; doi:10.1371/journal.pdig.0001611)
Supplement: Supplementary file 1 [file pdig.0001611.s001.docx]

## **S1 Appendix**

| **Patient** | **Diagnosis** | **Surgery** | **Age [years]** | **BSA [mm^2^]** | **SaO_2_ [%]** | **PSCPC [mmHg]** | **ICU Length [days]** | **Hospital Length [days]** |
| --- | --- | --- | --- | --- | --- | --- | --- | --- |
| **P1** | DORV+TGA | Norwood | 0.64 | 0.41 | - | - | 3 | 32 |
| **P2** | HLHS | Norwood | 1.63 | 0.48 | 89 | 10 | 8 | 6 |
| **P3** | HLHS | Norwood | 2.01 | 0.50 | 86 | 14 | - | - |
| **P4** | SV+TA | Not-Norwood | 2.06 | 0.53 | 85 | 10 | 3 | 9 |
| **P5** | HLHS | Norwood | 2.15 | 0.50 | 90 | 16 | 2 | 13 |
| **P6** | HLHS | Norwood | 2.29 | 0.55 | 84 | 16 | 2 | 7 |
| **P7** | SV+TA | Not-Norwood | 2.52 | 0.61 | 87 | 7 | 1 | 23 |
| **P8** | PA/IVS | Not-Norwood | 2.63 | 0.50 | 94 | 12 | 7 | 9 |
| **P9** | HLHS | Norwood | 2.63 | 0.66 | 87 | 18 | 2 | 14 |
| **P10** | DILV+HLHS+TGA | Norwood | 2.85 | 0.59 | 83 | 13 | 3 | 7 |
| **P11** | HLHS | Norwood | 2.88 | 0.62 | 88 | 11 | 7 | 13 |
| **P12** | TA | Not-Norwood | 2.92 | 0.55 | 86 | 8 | 2 | 5 |
| **P13** | SV+DILV | Not-Norwood | 2.96 | 0.55 | 85 | 11 | - | - |
| **P14** | SV+TA | Norwood | 2.97 | 0.59 | 89 | 11 | 3 | 6 |
| **P15** | HLHS | Norwood | 3.01 | 0.60 | 86 | 12 | 3 | 7 |
| **P16** | PA/IVS | Not-Norwood | 3.03 | 0.57 | 92 | 11 | 2 | 5 |
| **P17** | HLHS | Norwood | 3.08 | 0.58 | 94 | 14 | 5 | 8 |
| **P18** | SV+DILV | Not-Norwood | 3.11 | 0.66 | 91 | 7 | 8 | 23 |
| **P19** | PA/IVS | Not-Norwood | 3.13 | 0.60 | 85 | 9 | 1 | 5 |
| **P20** | HLHS | Norwood | 3.40 | 0.70 | 92 | 10 | 2 | 23 |
| **P21** | TGA+DILV | Norwood | 3.41 | 0.63 | 92 | 9 | 2 | 5 |
| **P22** | PA/IVS | Not-Norwood | 3.49 | 0.65 | 90 | 13 | 3 | 7 |
| **P23** | SV+TA | Not-Norwood | 3.52 | 0.64 | 84 | 12 | 1 | 4 |
| **P24** | HLHS | Norwood | 3.60 | 0.58 | 88 | 14 | 3 | 6 |
| **P25** | HLHS | Norwood | 3.69 | 0.58 | 86 | 7 | 3 | 8 |
| **P26** | TA+VSD+ASD | Not-Norwood | 4.10 | 0.56 | 88 | 8 | 2 | 12 |
| **P27** | DORV+TGA | Not-Norwood | 4.72 | 0.76 | 86 | 14 | 2 | 7 |
| **P28** | DORV+HLHS+MA | Norwood | 4.79 | 0.64 | 84 | 13 | 2 | 7 |
| **P29** | SV+TA | Not-Norwood | 5.32 | 0.72 | 82 | 18 | - | - |
